# Systematic review and meta-analysis of anti-thymocyte globulin dosage as a component of graft-versus-host disease prophylaxis

**DOI:** 10.1371/journal.pone.0284476

**Published:** 2023-04-18

**Authors:** Joice Zuckermann, Bruno Mateus de Castro, Thiago Almirante Cunha, Alessandra Paz, Leila Beltrami Moreira

**Affiliations:** 1 Graduate Program in Pharmacology and Therapeutics, Universidade Federal do Rio Grande do Sul, Porto Alegre, Brazil; 2 Hospital de Clínicas de Porto Alegre, Porto Alegre, RS, Brazil; 3 School of Medicine, Universidade Federal do Rio Grande do Sul, Porto Alegre, RS, Brazil; 4 Nursing School, Universidade Federal do Rio Grande do Sul, Porto Alegre, RS, Brazil; The University of Texas MD Anderson Cancer Center, UNITED STATES

## Abstract

Rabbit anti-thymocyte globulin (ATG) has been used in allogeneic hematopoietic stem cell transplantation (Allo-HSCT) for graft-versus-host disease (GvHD) prophylaxis. Since the best dose has not been defined yet, this study aimed to determine the efficacy and safety of different doses of ATG in Allo-HSCT. Data sources were MEDLINE/PUBMED, EMBASE, Cochrane Library, Web of Science, LILACS, and SciELO. Studies were eligible when comparing doses of ATG. The higher dose was in the intervention group. A total of 22 articles (2002–2022) were included. Higher doses (4–12 mg/kg) of ATG-T reduced the incidence of grade III-IV acute GvHD (RR 0.60; 95%CI 0.42–0.84) and limited chronic GvHD (RR 0.64 95%CI 0.45–0.92) compared with lower doses (2–7.5 mg/kg). Higher doses increased the Epstein-Barr virus (RR 1.90 95% CI 1.49–2.42) and *Cytomegalovirus* reactivation (RR, 1.30; 95% CI 1.03–1.64). Relapse rates were higher in the higher dose group (RR 1.34, 95% CI 1.07–167). The ATG-T dose ≥7mg/kg versus the lower dose showed a number needed to treat 7.4 for acute GvHD III-IV, with a number to harm of 7.7 for relapse at one year in the higher dose group. A dose lower than 7 mg/kg suggests a better risk-benefit ratio than a higher one. Well-designed RCT is needed to define the best risk-benefit doses.

**Trial registration:** Trial registration number: PROSPERO: https://www.crd.york.ac.uk/prospero/display_record.php?ID=CRD42020173449.

## Introduction

Graft-versus-host disease (GvHD) is an important factor that contributes to mortality and morbidity after allogeneic hematopoietic stem cell transplantation (HSCT). Rabbit anti-thymocyte immunoglobulin (rATG) has been used in HSCT conditioning regimens/prophylaxis as a strategy for in vivo depletion of T lymphocytes to prevent GvHD. The rATG products are polyclonal anti-thymocyte immunoglobulin G (IgG) antibodies derived from rabbits after immunization with human thymocytes or a T-lymphoblast cell line.

Meta-analysis of randomized clinical trials (RCT) of unrelated-donor HSCT has demonstrated the effectiveness of ATG in the prophylaxis of both acute and chronic GvHD in contrast with the non-use of ATG [1]. However, the incidence of *Cytomegalovirus* (CMV) and Epstein-Barr virus (EBV) reactivation was significantly higher in the group that received ATG compared to the control group.

In addition, the dosage and the types of ATG (Fresenius/ATLG/ATG-F or Thymoglobulin / ATG-T) used have been quite heterogeneous among transplant centers. Therefore, the dose with the best risk-benefit ratio in HSCT is not established yet. This systematic review aims to assess the impact of using higher total doses of ATG in comparison to lower total doses on the outcomes of acute and chronic GvHD, as well as on the incidence of post-transplant infections (CMV and EBV), risk of graft failure, relapse of primary disease, and mortality in allogeneic HSCT.

## Methods

For the systematic review, the PICO framework was used. The sample consists of patients subjected to allogeneic HSCT who used rATG as a strategy for T cell depletion in GvHD prophylaxis. The intervention was a higher dose of ATG. The outcomes are incidence of acute (grade II-IV and grade III-IV) or chronic (global, limited, or extensive) GvHD; post-transplant CMV reactivation; EBV reactivation (or EBV-associated lymphoproliferative disorder); graft failure (primary or secondary); relapse of primary disease; and transplant-related mortality.

The search was carried out in MEDLINE/PUBMED, EMBASE, Cochrane Library, Web of Science, LILACS, and SciELO databases, from 3/21/2020 to 3/01/2022, with no date limit. The search terms used were: “bone marrow transplantation,” “hematopoietic stem cell transplantation,” “haematopoietic stem cell transplantation,” “hematopoetic stem cell transplantation,” “antithymocyte globulin,” “globulin antithymocyte,” “immunoglobulin antithymocyte,” “antithymocyte antibody,” “graft versus host disease,” “graft vs host disease,” “graft vs host reaction,” “gvhd disease,” “gvhd.” The supporting information shows the search strategy employed in each database (S1 Table). The search also encompassed the reference section of the selected studies.

The inclusion criteria were comparative clinical studies in HSCT with related (HLA-matched or haploidentical) or unrelated (HLA-matched and HLA-mismatched) patients of all ages (including children), who used rATG (ATG-F or ATG-T) as a strategy for T-cell depletion in GvHD prophylaxis. The study should compare groups receiving different doses of ATG. There were no restrictions concerning language, follow-up time, or date limit. Exclusion criteria were not defined to enlarge the search. Two independent researchers selected the articles by title and abstract and read them fully and extracted the data. The data were recorded on a spreadsheet by each researcher. After comparison, disagreements were resolved by consensus. The data sought were author, period of the study, number of participants per group, sex, age, type of ATG, ATG group dose, diagnosis, stem cell source, donor, HLA compatibility, conditioning regime, and the outcomes. The tools selected to assess the quality of the studies were RoB 2 (risk of bias tool) [2] and ROBINS-I (Risk of Bias in Non-randomized Studies—of Interventions) [3] for the selected outcomes. The risk of publication bias was investigated by funnel plot and the meta-analysis was performed in RevMan 5.3. Heterogeneity was considered low for I^2^ ≤ 25%, moderate for I^2^ > 25% and ≤75%, and high for I^2^ > 75%. Subgroup analyses were performed according to the design of the studies, namely observational studies and randomized clinical trials, separated per type of ATG-T or ATG-F. Additionally, sensitivity analysis was conducted including only randomized studies in which the lower dose defined in each study was < 7 mg/kg and the higher dose ≥ 7mgKg, to compare studies without low and high doses overlap. The measure of association used in all outcomes was the relative risk with a 95% confidence interval. Data were analyzed using a random-effects model.

## Results

### Studies selection

**Fig 1:**
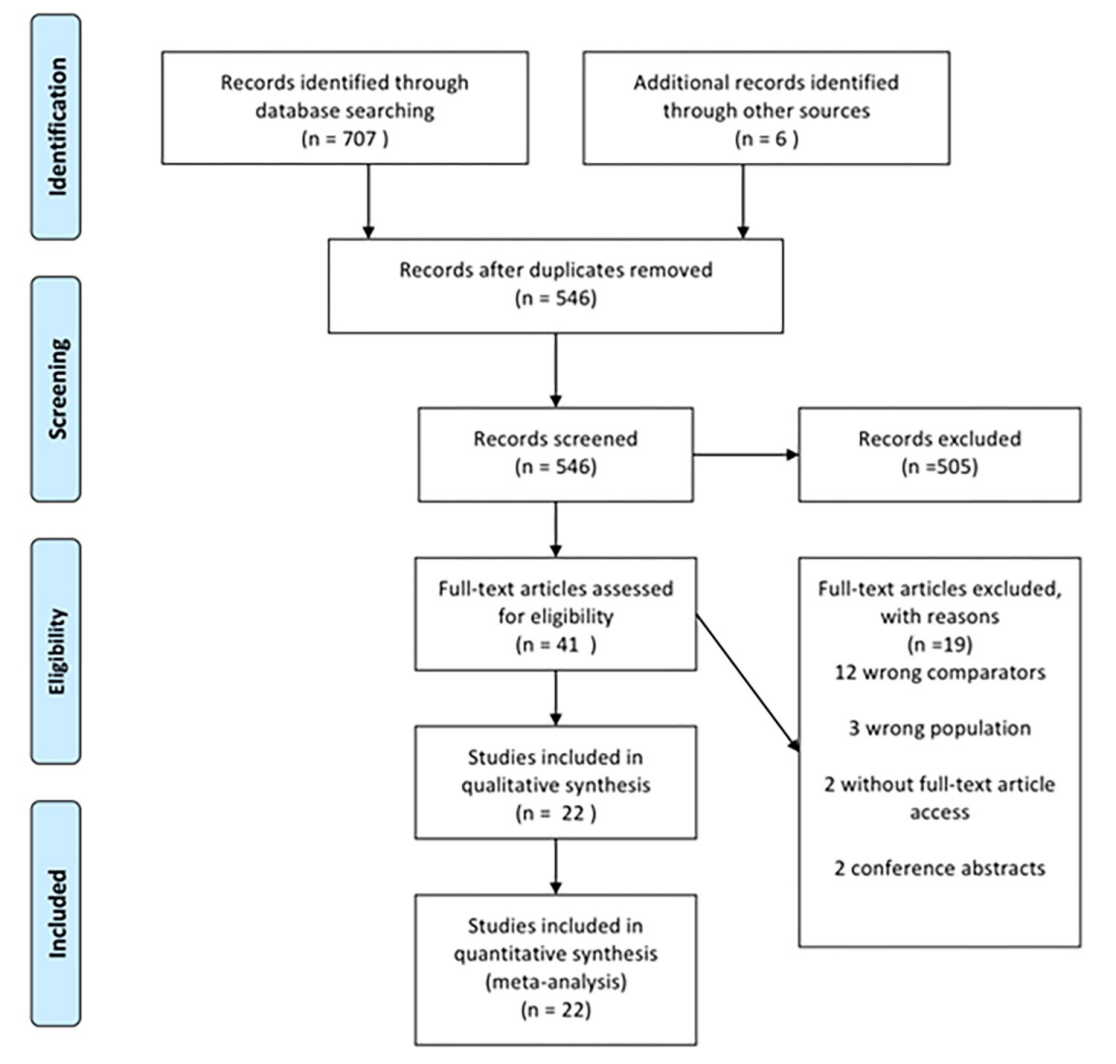
Study flowchart.

The flowchart illustrates the studies selection (Fig 1). A total of 707 articles were retrieved from the MEDLINE/PUBMED, EMBASE, Cochrane Library, Web of Science, LILACS, and SciELO databases, and six from the reference sections of the studies. After excluding duplicates, titles and abstracts were evaluated, resulting in 45 articles for eligibility assessment, and 22 were selected. Studies that showed at least two groups comparing outcomes for different doses of ATG were included. Bacigalupo 2001 [4] and Bacigalupo 2006 [5] were excluded because even using different doses of ATG, each group was randomized and compared with the group that did not receive ATG. The studies of Wang Y, 2014 [6] and Chang Y J, 2017 [7] have the same sample but report different primary outcomes: the first one reported aGvHD and cGvHD, and the second one only cGvHD. Thus, in the cGvHD analysis, the study performed by Chang Y J, 2017 [7] was considered since it includes the outcomes from Wang Y, 2014. Yu G P 2012 [8], Choi, Y 2016 [9], and Park, 2017 [10] were excluded due to the impossibility of fully accessing the study, even after requesting to the authors via the ResearchGate.net.

### Study characteristics

Table 1 shows the characteristics of the studies. The date of publication of the selected studies ranged from 2002 to 2021. A total of six were randomized clinical trials [6,7,11–14] and 16 were observational studies [15–30] (12 retrospective and 4 prospective). They were conducted in the United States, Europe (France, Germany, the Netherlands, Italy, and Switzerland), and Southeast Asia (China, Japan, and South Korea).

**Table 1:** Studies comparing ATG doses characteristics.

Author/ period	Study design	N Higher ATG/Lower ATG doses	Sex n (%) M/F All sample	Median age Higher ATG/ Lower ATG doses	Diagnosis	Stem cell sources	Donor	HLA compatibility	Conditioning regime	ATG type	ATG higher total dose (mg/Kg b.w.)	ATG lower total dose (mg/Kg b.w.)	Other immunosuppressive
**Ayuk F, 2008 [15]** Jul 1997—Oct 2006	Cohort Retrospective	49 / 34	40 (48) / 43 (52)	35/37	ALL; AML; MDS; CML	BM; PB	UD	no mismatch, except HLA-C	Myelo-ablative	ATG-F	60	30	CSA; MTX
**Bashir Q, 2012 [11]** Jan 2004—Jan 2007	RCT	15 / 5	11 (55) / 9 (45)	60/60	AML; MDS	BM; PB	UD	no mismatch HLA-A, HLA-B, HLA-DRB1, and HLA-DQB1	RIC	ATG-T	7.5	4.5	TAC; MTX
**Butera S, 2021 [27]** Jan 2005—Dec 2016	Cohort Retrospective	198 / 197	216 (55) / 179 (45)	50.4 / 52.4	ALL; AML; MDS; MPN; LPD	BM; PB	UD	mismatch and no mismatch	Myelo-ablative RIC	ATG-T	6–7.5	5	CSA + MTX CSA + MMF
**Chang, Y.J. 2017 [7]** Dec 2010—May 2012	RCT	112 / 112	153 (68) / 71 (32)	28.5 / 27	AML; ALL; CML; MDS	BM; PB	Haplo	mismatch HLA-A, HLA-B, HLA-DR	Myelo-ablative	ATG-T	10	6	NR
**Devillier R, 2018 [16]** Jan 2000- Feb 2013	Cohort Retrospective	39 / 195	NR	55 / 55	AML	PB	RD	no mismatch	RIC	ATG-T	7.5 (6–10)	5 (2–5.5)	CSA
**Hamadani M, 2009 [17]** Jan 2006—Dez 2008	Cohort Prospective	39 / 33	49 (68) / 23 (32)	56 / 55	MDS; AML; CLL; NHL	BM; PB	UD RD	mismatch and no mismatch HLA-A, B, C DRB1	RIC	ATG-T	7.5	6.0	TAC; MTX

**Hatanaka K, 2012 [18]** Apr 2001- Dec 2006	Cohort Retrospective	21 / 31	54 (63) / 32 (37)		31 / 31	AML; MDS; CML; NHL; MM; AML; myeloproliferative diseases; primary immunodeficiency hereditary metabolic diseases	BM	UD Haplo	mismatch and no mismatch	Myelo-ablative; RIC; Non-Myelo-ablative	ATG-F	>10	<10	TAC ± MTX; CSA±MTX; TAC ± MTX + prednisolone
**Issa H, 2019 [19]** Oct 2007 –Sep 2016	Cohort Retrospective	216/141	214 (60) / 143 (40)	59/53		AML; MDS; NHL; HD; CLL; other	BM; PB	UD	mismatch and no mismatch	RIC	ATG-T	6	4.5	TAC+MTX;TAC+MMF; CSA/MMF or TAC/sirolimus CSA/MTX and CSA/MMF
**Kang M H, 2021 [28]** Apr 2009—Sep 2018	Cohort Retrospective	118/78	123 (63) / 73 (37)	10.5 / 7.7		AML, ALL, CML, JMML	PB	UD Haplo	mismatch and no mismatch	Myelo-ablative RIC	ATG-T	7.5 or 10	3.75 or 5	CSA + MTX
**Kim T,** 2019 **[20]** 2005–2015	Cohort Retrospective	249/103	210 (60) / 142 (40)	46 / 46		AML; ALL; BAL; SMD	BM; PB	Haplo UD	no mismatch A, B, C, and DR	Myelo-ablative RIC	ATG-T	9 or 12	2.5–7.5	NR
**Li C, 2012 [21]** Dec 2008—Dec 2010	Cohort Prospective	32/32	43 (67) / 21 (33)	6.25 / 7.31		severe beta thalassemia	BM; PB	UD RD	mismatch and no mismatch	Cy+ Bu+Flu+ thiotepa	ATG-F	10	7.5	CSA; MTX
**Lin R, 2019 [12]** Jun 2013—Jan 2016	RCT	205/ 203	278 (68) / 130 (32)	26 / 28		AML,ALLpH+, ALLpH-, BAL	BM; PB	Haplo	mismatch	Myelo-ablative	ATG-T	10	7.5	CSA; MTX; MMF

**Locatelli F, 2017 [14]** Jan 2008—Set 2012	RCT	84 / 88	108 (63) / 64 (37)	9 / 9	AML; CML; MDS NHL	BM; PB	UD	mismatch and no mismatch HLA-A, B, C, DRB1	Myelo-ablative	ATG-F	30	15	CSA; MTX
**Meijer E, 2003 [22]** Jan 1999—May 2002	Cohort Retrospective	52 / 22	NR	34 / 35–37	AML,ALL,CML ASA, Other	BM; PB	UD	mismatch and no mismatch	RIC	ATG-T	8	6 or 4	CSA; MTX
**Mohty M, 2003 [23]** Apr 1998—Jun 2002	Cohort Retrospective	46 /55	NR	50 / 50	AML; MDS; ALL; CML; CLL; HD; NHD; MM; non-hematological malignancies	BM; PB	RD	no mismatch HLA-A-, HLA-B-, and HLA-DR	RIC	ATG-T	10 or 7.5	2.5	CSA
**Park S S, 2019 [24]** May 2004—Mar 2016	Cohort Retrospective	All: 324 ATG = 257 154 / 103	218 (67) / 106 (33)	49 / 49	MDS; CML; AML from MDS	BM; PB	RD UD Haplo	mismatch and no mismatch on UD	RIC; Myelo-ablative	ATG-T	7.5–10	2.5–5	TAC (NAP or HAPLO) or CSA (AP) + MTX
**Remberger M, 2004 [25]** Nov 1989—Jun 2003	Cohort Prospective	74 / 88	90 (55) / 72 (44)	27 / 27	AML; MDS; ALL; CML; other malignancies	BM; PB	UD	no mismatch HLA-A, -B, and–DR	Myelo-ablative	ATG-T	8 or 10	4 or 6	CSA+MTX; CSA+prednisoloneCSA+MMF
**Sallas Q M, 2021 [29]** Aug 2016—Feb 2020	Cohort Retrospective	60 /3 5	56 (60) / 39 (40)	56 / 58	AML, MDS, ALL, CLL, MPN, BPDCL	PB	Haplo	mismatch	Myelo-ablative RIC	ATG-T	4.5	2	CY; CSA

**Schleuning M, 2003 [26]** Sep 1990—May 2001	Cohort Pro-spective	66 / 31	57 (59) / 40 (41)	36 / 36	CML	BM	UD	mismatch and no mismatch HLA-A, HLA-B, HLA-DRB1 and HLA-DQB1	Myelo-ablative	ATG-F	60, 75, 80, 120	50,40 30,20	CSA; MT
**Toor A A, 2015 [13]** 2009–2013	RCT	22 / 19	26 (63) / 15 (37)	57 / 57	MM, NHL, AML, MDS, CLL, PLL	BM; PB	RD UD	mismatch and no mismatch HLA-A, -B, -C, and -DRB1	RIC	ATG-T	7.5	5.1	TAC+MMF
**Wang Y, 2014 [6]** Dec 2010- May 2012	RCT	112 / 12	153 (68) / 71 (32)	28.5 / 27	AML, ALL, CML, MDS	BM; PB	Haplo	mismatch	Myelo-ablative	ATG-T	10	6	CSA; MTX; MMF
**Zhou X, 2020 [30]** Mar 2013—Dec 2018	Coorte Retro-spective	57 / 59	144 (66) / 72 (33)	26 / 28	AML, ALL, CML, MDS, MAL	BM; PB	Haplo	mismatch	Myelo-ablative RIC	ATG-T	9	6	TAC+MTX +MMF + antiCD25 monoclonal antibody

ALL: Acute lymphoblastic leukemia; AML: Acute myeloid leukemia; ATG: Anti-thymocyte immunoglobulin; BAL: Biphenotypic acute leukemia; BM: Bone marrow; CLL: Chronic lymphocytic leukemia; CML: Chronic myeloid leukemia; CsA: Cyclosporine; Haplo: Haploidentical; HL: Hodgkin’s lymphoma; HSCT: Hematopoietic stem cell transplantation; M/F: Male/female; MDS: Myelodysplastic syndromes; MM: Multiple myeloma; MMF: Mycophenolate mofetil; MTX: Methotrexate; NHL: Non -Hodgkin’s lymphoma; NR: Not reported; PB: Peripheral blood; Ph+ALL: Philadelphia Chromosome-positive acute lymphoblastic leukemia; Ph-ALL: Philadelphia Chromosome-negative acute lymphoblastic leukemia; PLL: Prolymphocytic leukemia; R: Related; RIC: Reduced-intensity conditioning; SAA: Severe aplastic anemia; TAC: Tacrolimus; UR: Unrelated.

The median age of patients reported in the groups who received the higher doses of ATG-T ranged from 26 to 60, and ATG-F from 6 to 36 years, respectively. In the groups that received the lower doses of ATG-T and ATG-F, the median age ranged from 27 to 60 and 7 to 37 years, respectively. All patients had been diagnosed with malignant or non-malignant hematological disorders. In Mohty, M. 2003 [23], 30% of the sample was diagnosed with non-hematological malignancies (breast cancer, renal cell cancer, ovarian cancer, melanoma, and five not reported cancers). In Issa, H. 2019 [19], 22% of the sample presented unspecified diagnoses, and in Meijer, E. 2003 [22], 25.8% of the sample received other diagnoses (not specified). In Remberger, M. 2004 [25], 2.19% of the sample was diagnosed with other malignancies (not specified). All patients underwent allogeneic hematopoietic stem cell transplantation. The HSCT modalities were related (HLA-matched and HLA-mismatched), haploidentical, and unrelated. There was always a representative of each modality in both higher and lower dose groups, yet not necessarily in the same proportion.

The hematopoietic cells were collected from bone marrow (BM) and peripheral blood (PB) in all studies, except in Hatanaka, 2012 [18] and Schleuning, M. 2003 [26] (only BM), and Devillier, R. 2018 [16] (only PB). In the studies by Bashir, Q. 2012 [11] and Mohty, M. 2003 [23], PB cells predominated in the lower dose group (73.3% and 80%, respectively), while BM cells were the majority in the higher dose group (both 91%). In Remberger, M. 2004 [25], the use of BM predominated in the higher dose group (82%), but in the lower dose group, patients received similar amounts of PB and BM cells. In Park, S. S. 2019 [24] and Li, C. 2012^21^, the use of PB preponderated (96.3% and 93.7%, respectively). Conversely, in Meijer, E. 2003 [22], the number of BM cells was more significant (95.9%).

The conditioning regimens varied according to the primary diseases. For GvHD prophylaxis, the immunosuppressants used were MTX, CsA, Tacrolimus, MMF, and Prednisolone. The supportive therapies employed, considering the spectrum of antimicrobials for viruses (especially EBV and CMV), bacterial agents, and fungi included Acyclovir, Valacyclovir, Ganciclovir, Valganciclovir, Foscarnet, Rituximab, SMT-TMP, Dapsone, Amphotericin B, Colistin, Cephalothin, Levofloxacin, Ciprofloxacin, Vancomycin, Fluconazole, Posaconazole, Itraconazole, Cotrimoxazole, Pentamidine, and Micafungin.

Due to differences in manufacturing procedures, specificities, and the number of antibodies, the studies used two categories of anti-thymocyte globulin: ATG-Fresenius (ATG-F) and ATG-Gemzyme (ATG-T). A total of five studies (one RCT and four observational) employed ATG-F, and 17 studies used ATG-T (Thymoglobulin) (5 RCTs and 12 observational). The analysis was stratified by ATG-T and ATG-F. The included studies considered different doses as high or low. Among the studies included, the higher dose of ATG-T was 4–12 mg/kg, and the lower was 2–7.5 mg/kg. The ATG-T was administered between −9 and −1 pre-transplant days, considering the studies that reported this information. The highest dose of ATG-F ranged >10–120 mg/kg, and patients received it between −5 and −1 pre-transplant days. The lower dose of ATG-F ranged <10–50 mg/kg.

### Risk of bias in the selected studies

The risk of bias is graphically summarized in the supporting information (S1 Fig). All observational studies showed a high risk for selection, detection, and performance bias. The randomized clinical trials had a low risk for selection bias, except for Bashir, Q 2012 [11] and Toor, A 2015 [13]. Bashir, Q 2012 [11] used an adaptive Bayesian randomization model and was considered at high risk. Toor, A 2015 [13] reported insufficient information regarding allocation concealment. As for performance bias, all open-label RCTs were considered high risk. Detection bias was considered low risk in three RCTs because they used either a blinded committee to evaluate the outcomes or blinded outcome evaluators to analyze the treatment received. Cases of insufficient information about blinding of the outcome evaluators were considered an unclear risk for detection bias. Some studies did not report the outcome of chronic GvHD for all patients initially included, and thus were considered at high risk for attrition bias. Selective outcome reporting was considered low risk in all studies.

### Publication bias for ATG-T

The funnel plot assessed publication bias for the outcomes of aGvHD (grade II-IV and grade III-IV) and cGvHD (global, limited, and extensive). Visual inspection suggests the risk of publication bias concerning extensive cGvHD and aGvhD for ATG-T. See supporting information for details (S2 Fig).

### ATG-T outcomes

#### Acute GvHD (grades II-IV and III-IV)

**Fig 2:**
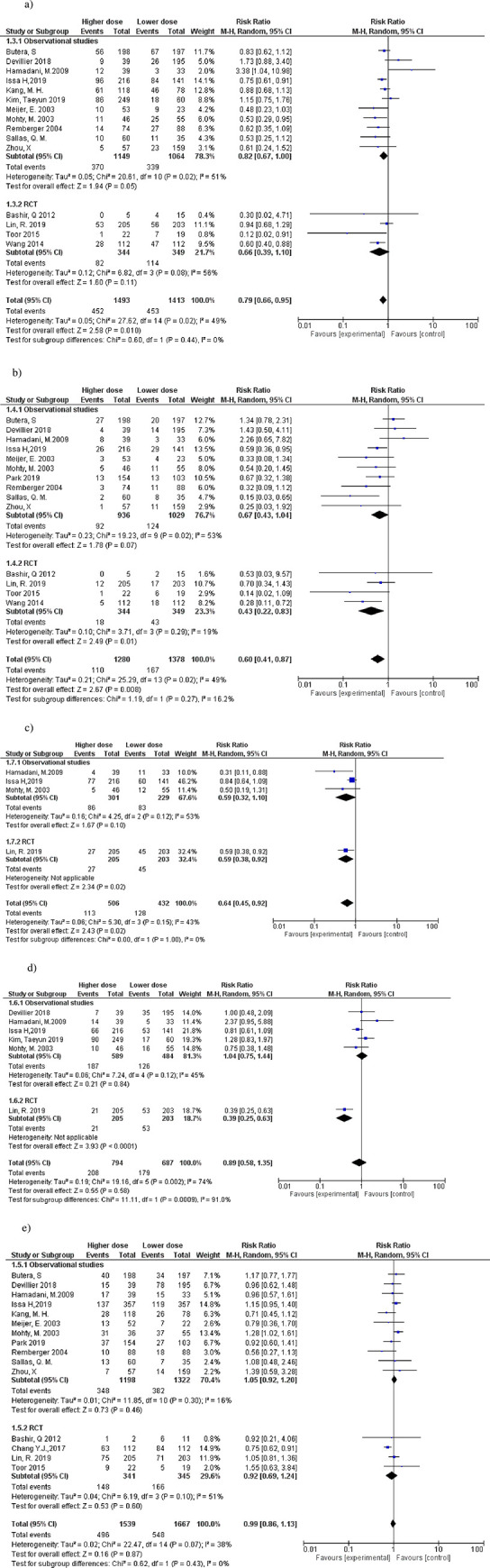
Forest plots for the comparison of acute and chronic GvHD between the higher and the lower doses of ATG-T. a) II-IV Acute GVHD. b) III-IV Acute GVHD. c) Limited chronic GVHD. d) Extensive chronic GVHD. e) Global chronic GVHD. The effect measure (relative risk) of each study is indicated by blue boxes (size proportional to the importance of the study in the meta-analysis), with lines indicating a 95% confidence interval (95% CI). The black diamonds represent the summary effect measure and the 95% confidence interval.

In 3,563 patients who used ATG-T, the incidence of aGvHD was a dichotomous variable: grades II-IV and III-IV (Fig 2(A) and 2(B)). Among the studies that reported GvHD as an outcome, the definition of acute GvHD followed the classic Glucksberg scale [31]. Only one study [24] used the Mount Sinai International Consortium [32], and three [12,19,20] used the Keystone Consensus Conference on Acute GvHD Grading [33].

The incidence of grade II-IV aGvHD was lower in the groups that used the higher doses of ATG-T (RR, 0.79; 95%CI 0.66–0.95 I^2^ = 49%), as shown in Fig 2(A). The incidence of grade III-IV aGvHD (Fig 2(B)) was also lower for patients in the higher dose ATG-T group (RR 0.60; 95%CI 0.41–0.87 I^2^ = 49%).

#### Chronic GvHD (global, limited, or extensive)

The incidence of cGvHD (limited, extensive, or global) was assessed as a dichotomous variable (Fig 2(C), 2(D) and 2(E)). The definition of cGvHD among the studies that reported this outcome predominantly followed the Seattle [34] or the modified Seattle scale [35], but the NIH (2005) [36] and NIH (2014) criteria were also used [37]. All outcomes in our study were reported according to the Seattle scale, as it was the most frequently utilized in the studies. In studies that followed the NIH classification, we considered only global GvHD. The global GvHD outcome included the total sum of events in the studies that reported both limited and extensive GvHD.

The highest doses of ATG-T significantly reduced the risk of limited cGvHD (RR 0.64 95%CI 0.45–0.92; P = 0.02 and I^2^ = 34%) (Fig 2(C)). The incidence of extensive cGvHD showed no significant difference between groups for ATG-T (Fig 2(D)). Data on the incidence of global GvHD outcomes in patients using ATG-T were extracted from 18 studies including 3,206 patients (1,539, ATG-T higher dose; 1,667, ATG-T lower dose). There was no significant difference between the groups in studies with ATG-T (Fig 2(E)).

#### Incidence of *Cytomegalovirus* (CMV) reactivation after transplantation

**Fig 3:**
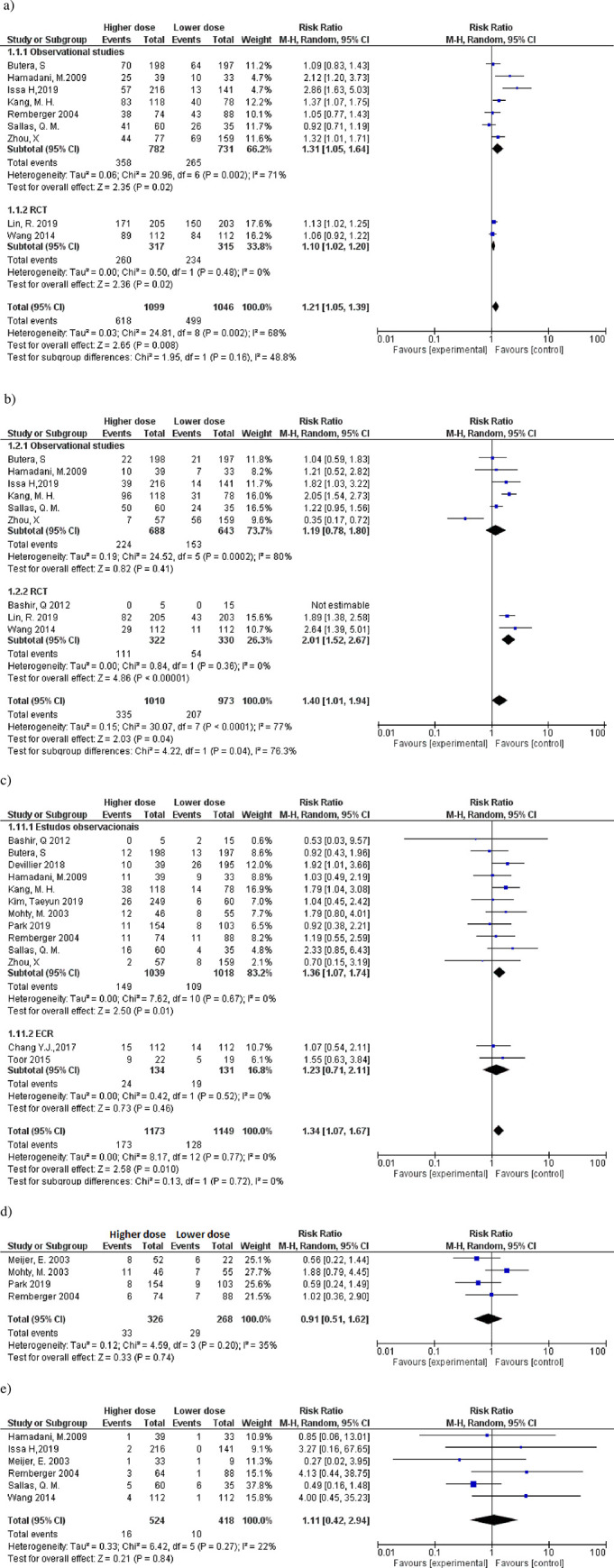
Forest plot for the comparison of the incidence of *Cytomegalovirus* (CMV) reactivation after transplantation; EBV reactivation (or EBV—associated lymphoproliferative disorder); graft failure (primary or secondary); relapse of the primary disease, and transplant-related mortality with the higher and lower doses of ATG-T. a) CMV reactivation. b) EBV reactivation or lymphoproliferative disorder associated with EBV. c) Primary disease recurrence rate (relapse) corrected for 1 year. d) Transplant-related mortality corrected for 1 year. e) Primary or secondary graft failure. The effect measure (relative risk) of each study is indicated by blue boxes (size proportional to the weight of the study in the meta-analysis), with lines indicating a 95% confidence interval (95% CI). The black diamond represents the summary effect measure and the 95% confidence interval.

The risk of CMV reactivation (Fig 3(A)) increased when higher doses of ATG-T are used (RR, 1.21; 95%CI 1.05–1.39; I^2^ = 68%). Notably, the risk is reduced when the randomized clinical trials were summarized (RR, 1.10; 95% CI 1.02–1.20; I^2^ = 0%).

### Incidence of EBV reactivation (or EBV-associated lymphoproliferative disorder)

Considering nine studies (Fig 3(B)), EBV reactivation increased with higher doses of ATG-T (RR 1.40; 95%CI 1.01–1.94; p <0.00001, I^2^ = 77).

### Recurrence of primary disease

The recurrence and relapse of the primary diseases were considered equivalent outcomes. The incidence of relapse was higher in the higher ATG-T dose group (RR 1.34, 95% CI 1.07–1.67, I^2^ = 0%) (Fig 3(C)). Table 1 describes all primary diseases evaluated in this outcome. Studies with severe aplastic anemia, beta-thalassemia, primary immunodeficiency disorders, myeloproliferative disorders, and primary metabolic disorders did not report the recurrence of the primary disease.

### Transplant-related mortality

Only four studies reported transplant-related mortality. There was no significant difference between the effects of higher and lower doses of ATG-T on transplant-related mortality (Fig 3(D)).

### Graft failure (primary or secondary)

A total of six studies using ATG-T reported graft failure (Fig 3(E)); two studies reported secondary failure [17,19] and showed no difference between higher and lower doses. This outcome was not analyzed by observational studies or randomized clinical trials stratification due to the lack of studies. Only one study [15] reported no graft failure events. There was no difference between doses regarding the conditioning modality.

### ATG-F outcomes

The forest plots for ATG-F outcomes are presented in supporting information (S3 Fig).

### Sensitivity analyses

**Fig 4:**
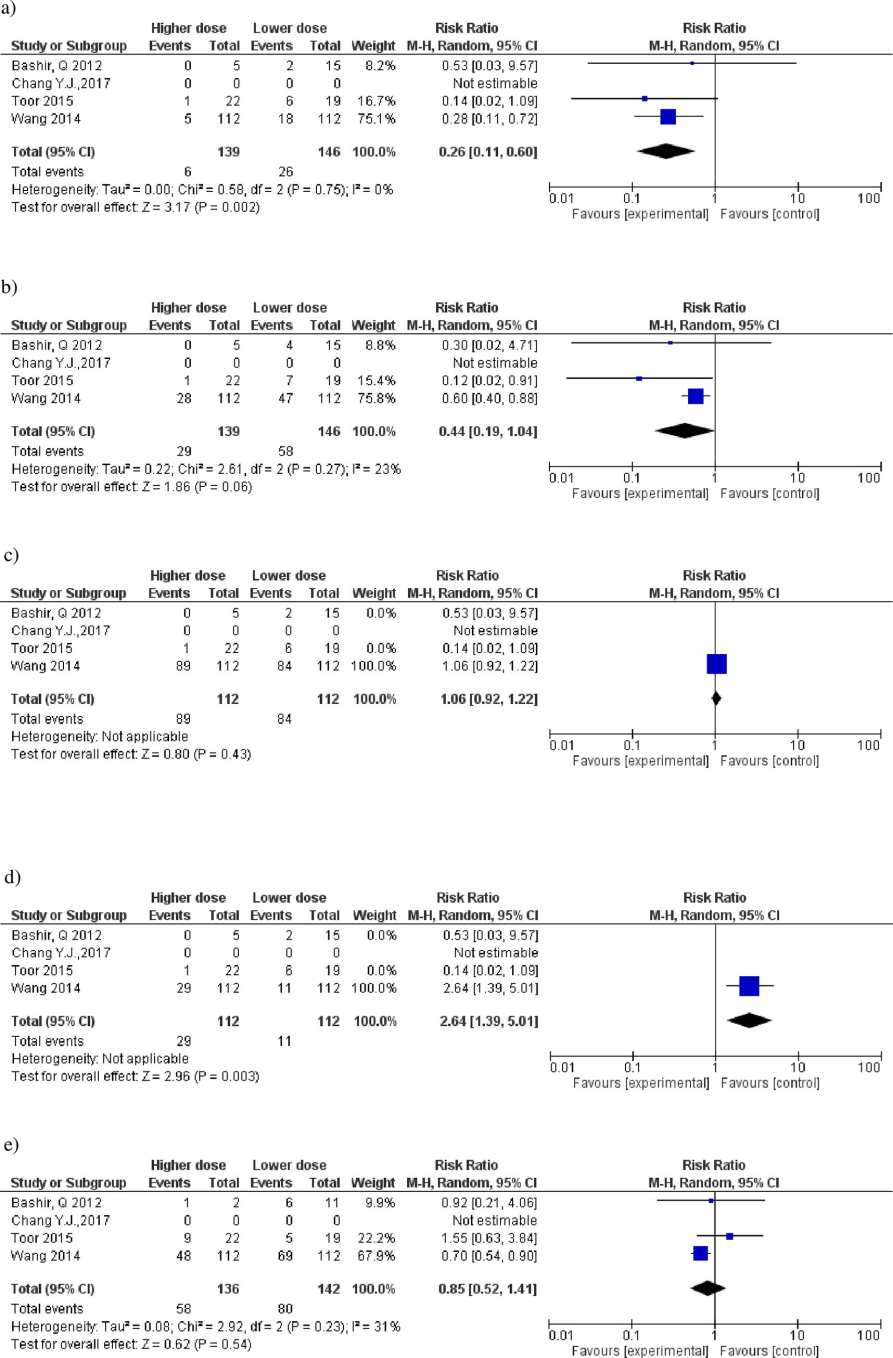
Forest plot for the comparison of ATG-T doses <7 mg/kg with ≥7 mg/kg. a) III-IV acute GVHD. b) II-IV acute GVHD. c) CMV reactivation. d) EBV reactivation or EBV-associated lymphoproliferative disorder. e) Global chronic GVHD. f) Recurrence rate of primary disease (relapse) corrected for 1 year. The effect measure (relative risk) of each study is indicated by blue boxes (size proportional to the weight of the study in the meta-analysis), with lines indicating a 95% confidence interval (95% CI). The black diamond represents the summary effect measure and the 95% confidence interval.

The post hoc sensitivity analysis included only randomized clinical trials in which groups received higher ATG-T doses ≥ 7 mg/kg versus lower doses < 7 mg/kg without dose overlap among studies. There was a 74% risk reduction of acute III-IV GvHD (RR 0.26 95%CI 0.11–0.60) in the group that used doses ≥ 7 mg/kg (Fig 4(A)). There was a tendency of benefit for acute II-IV GvHD (RR 0.44 95 CI 0.19–1.04) (Fig 4(B)). The risk increased by 80% for relapse at one year (RR 1.80 95%CI 1.13–2.89) (Fig 4(C)) and 64% for EBV reactivation or EBV-associated lymphoproliferative disorder (RR 2.64 95%CI 1.39–5.01) in the higher dose group (Fig 4(E)).

The sensitivity analysis for the type of transplantation showed that patients subjected to unrelated transplantation (HLA-matched and HLA-mismatched) who used the higher dose of ATG-T had a lower incidence of both II-IV aGvHD (RR 0.71; 95%CI 0.59–0.85; I^2^ = 0%) and III-IV aGvHD (RR 0.52, 95% CI 0.34–0.79; I^2^ = 0%) (S4(A) and S4(B) Fig). Considering the conditioning modality, the meta-analysis suggests that the higher doses are beneficial to patients undergoing myeloablative conditioning for II-IV aGvHD prevention (RR 0.73; 95%CI 0.53–1.00; I^2^ = 45%), as well as for the incidence of III-IV aGvHD (RR 0.44; 95%CI 0.24–0.83; I^2^ = 26%) (S4(C) and S4(D) Fig). There was no difference between higher and lower doses in patients subjected to reduced-intensity conditioning (RIC) in the prevention of aGvHD, while the risk of global cGvHD was significantly increased (RR, 1.15; 95%CI 1.01–1.31; I^2^ = 0%) in the higher doses of ATG-T (S4(C), S4(D) and S4(E) Fig).

The sensitivity analysis by type of transplantation showed that the risk of CMV reactivation with the higher doses was among haploidentical HSCT (RR, 1.10; 95% CI 1.0–1.21; I^2^ = 29%) (S5(A) Fig). Regarding both types of transplantation and the conditioning modality, the risk of reactivation with the higher dose remained in the myeloablative conditioning group (RR, 1.10; 95% CI 1.02–1.19; I^2^ = 0%), particularly in the RIC group (RR, 2.46; 95% CI 1.65–3.68; I^2^ = 0%) (S5 Fig).

Myeloablative conditioning maintained the greatest risk of EBV incidence (S5(C) Fig) with the higher doses of ATG-T (RR, 2.01; 95%CI 1.52–2.67; I^2^ = 0%). A tendency for greater risk also appeared in the RIC (RR, 1.60; 95%CI 1–2.57; I^2^ = 0%). The higher doses of ATG-T showed a greater risk of recurrence and relapse of the primary disease (S5(D) Fig) for RIC patients (RR, 1.53; 95%CI 1.05–2.23; I^2^ = 0%). There was no difference between doses in myeloablative conditioning.

Considering the timing of ATG infusion, the sub-group of the first infusion of ATG from day −5 to day −1 showed lower incidence in the higher dose group of both II-IV and III-IV aGvHD (RR, 0.39 95%CI 0.22–0.69; RR, 0.23 95%CI 0.10–0.51, respectively), while there was no difference in the sub-group of first infusion from day −9 to day −6 (RR, 0.74; 95%CI 0.55–1.00; RR, 0.66; 95%CI 0.38–1.15) (S6(A) and S6(B) Fig). However, the heterogeneity between the studies is intermediate (I^2^ = 50%; I^2^ = 56%, respectively).

## Discussion

This study aimed to evaluate the effectiveness and safety of higher versus lower total doses of ATG on GvHD prevention after allogeneic HSCT. A total of six RCTs and 16 observational studies (12 retrospective and four prospective) using ATG-T or ATG-F were analyzed. The meta-analysis showed that the higher total dose of ATG-T (6 to 12 mg/kg) is more beneficial than the lower dose (2 to 7.5 mg/kg) in the prevention of grade III-IV aGvHD and limited cGvHD. However, using the highest doses demonstrated a higher risk of CMV reactivation (high heterogeneity) and EBV or EBV-associated lymphoproliferative disorder.

The EBMT 2020 consensus [38] recommends a total ATG-T dose of 2.5–5 mg/kg in related HSCT and 4.5–6 mg/kg in unrelated HSCT, which are considered as the lower doses in our meta-analysis. The EBMT recommendation is supported by some studies, considering the risk of infection complications and benefits [39]. Deeg et al. [40] conducted a non-controlled intervention using doses of 4.5–6mg/kg of ATG in the BUCY protocol (busulfan and cyclophosphamide) in related and unrelated allogeneic HSCT to reduce the incidence of GvHD. By indirect comparison with a contemporary cohort without ATG, they concluded that doses of 4.6–6mg/kg of ATG supported the reduction of GvHD in allogeneic HSCT of peripheral cells. In our meta-analysis, the sub-group of four RCTs with 693 patients showed greater efficacy for higher doses than the recommended by EBMT in preventing III-IV acute GvHD (high grade of evidence) without difference for chronic GvHD. It showed both a 30% increase in CMV risk and twice EBV reactivation and an 80% increased risk for relapse at one year. However, there are few RCTs evaluating the risk of viral reactivation and primary disease recurrence.

A higher dose for unrelated HSCT recommended by EBMT 2020 consensus agrees with the sensitivity analysis. It showed that unrelated HSCT (HLA-matched and HLA-mismatched) benefit more from the highest ATG-T total doses to reduce the incidence of aGvHD (grade II-IV and grade III-IV), without heterogeneity among studies. There was no difference between doses regarding the incidence of global or extensive cGvHD, nor in general nor by type of HSCT. The reduction in the rate of limited cGvHD was significant for the higher doses group. However, it was impossible to perform sensitivity assessment by type of HSCT because of a lack of studies.

Regarding the safety of the intervention, the higher dose of ATG-T showed a greater risk of CMV reactivation and, especially, of EBV (or EBV-associated lymphoproliferative disorder) reactivation. In unrelated HSCT, sensitivity analysis detected no differences between higher and lower doses in CMV reactivation risk, which may be due to the high heterogeneity resulting from this sub-analysis (I^2^ = 91%). On the other hand, considering only randomized clinical trials, the meta-analysis of haploidentical studies maintained the risk with the higher dose. The lack of studies precluded sensitivity analysis on EBV outcomes.

The summarized risk in the stratified analysis by study design showed that the RR and 95%CI for observational studies are inside of the summarized RR and 95%CI for RCT. Despite the small number of patients included in the two RCTs, the agreement between the study design groups suggests a real risk. Primary or secondary graft failure and relapse of the primary disease showed no difference concerning total dose of ATG-T.

Only five studies compared different doses of ATG-F. The review from the Acute Leukemia Working Party of the European Society for Blood and Marrow Transplantation [41] recommends a 4.5 mg/kg–6 mg/kg total dose of ATG-T and 15–30 mg/kg of ATG-F for GvHD prophylaxis in peripheral blood-related HSCT. Further randomized clinical trials should be conducted to assess the best dose range of ATG-F, especially those including adults.

Concerning the prevention of grade III-IV aGvHD, the risk-benefit ratio estimated from the meta-analysis showed an absolute risk reduction of 4.7% and an increase of 6.8% in absolute risk of CMV reactivation and 13% in absolute risk of EBV (or EBV+LPD). The result is three cases of CMV reactivation, and six cases of EBV (or EBV+LPD) reactivation for two cases of aGvHD (grade III-IV) prevented. Considering the reduction in the absolute risk of limited cGvHD of 7.3%, one CMV reactivation and two EBV (or EBV+LPD) reactivations occur for each cGvHD prevented.

In the analysis including only RCTs comparing ≥ 7 mg/kg (high-dose) versus < 7 mg/kg (low-dose) groups, the number of patients needed to treat (NNT) is 7.4 for the outcome of acute GvHD III-IV, with a number needed to harm (NNH) of 7.7 for relapse at one year in the higher dose group. Based on these findings, we hypothesize that the administration of ATG-T total dose lower than 7 mg/kg has a better risk-benefit association. However, the RCT considered in the sensitivity analysis included patients with different clinical characteristics that must be considered for selecting the ATG personalized dose [42]. Even the use of a personalized dose may be less than utilitarian in some cases in which ATG may influence relapse rates. For example, the relapse rate may be unacceptably high in subjects with early-stage, good-risk AML or MDS undergoing RIC conditioning for transplantation with ATG prophylaxis.

Findings from Admiraal et al. [43] suggest that body weight above 50 kg does not affect the clearance of ATG in adults. Instead, it might correlate with the absolute lymphocyte count of the recipient before the first infusion of ATG. Therefore, the dosage of ATG based on body weight (mg/kg) might not be the most accurate strategy considering ATG pharmacokinetics. However, as the authors indicate, the approach of a fixed dose from a nomogram based on the recipient’s lymphocyte count must be addressed in prospective studies conducted in different HSCT settings and conditioning protocols to modify the current standard of care. No studies included in this review reported the absolute lymphocyte count before the first infusion of ATG.

The toxicity of ATG regarding poor T-cell recovery, leading to viral reactivations and relapse, is suggested to be related to in-vivo exposure of the graft to ATG [44]. Therefore, the timing of ATG may also affect outcomes. In the sensitivity analysis stratified per first ATG-T infusion day (from day −5 to day −1 and from day −9 to −6), comparing higher versus lower doses on each available outcome, reactivation was not different between doses in both strata, but the number of studies is small, and heterogeneity is high (I^2^: 90%), precluding any conclusion.

This systematic review with meta-analysis has limitations due to the significant number of observational studies and their high risk of bias. Also, the range of higher and lower doses used in the primary studies was quite variable. The diversity of diseases in the included studies and the therapeutic window for ATG may have contributed to different definitions for lower and higher dose cutoffs among studies. Third, the methods of CMV and EBV detection have changed over time, and the heterogeneous group of diseases included might impact relapse rate analysis. Finally, the number of administered doses can have significant effects on long-term transplant outcomes and complications and was not evaluated in this metanalysis.

## Conclusion and relevance

The results of this meta-analysis show the superiority of the higher doses in preventing GvHD and the greater risk of CMV and EBV infections and relapse. However, the low methodological quality of the studies and the lack of uniformity in the definition of high and low doses precludes a risk-benefit conclusion. Large and well-designed RCTs are needed to answer this relevant question.

## Supporting information

S1 Checklist PRISMA 2020 checklist. (DOCX)

S1 Fig Risk of bias. (DOCX)

S2 Fig Funnel plot for ATG-T. (DOCX)

S3 Fig Forest plots for ATG-F in each outcome. (DOCX)

S4 Fig Sensitivity analysis per type of transplantation. (DOCX)

S5 Fig Sensitivity analysis for CMV and EBV, and recurrence. (DOCX)

S6 Fig Sensitivity analysis per first ATG–T infusion day. (DOCX)

S1 Table Search methods and number of studies recovered. (DOCX)

S1 File (DOCX)
