# Supplementary figures and images for: Systematic review and meta-analysis of anti-thymocyte globulin dosage as a component of graft-versus-host disease prophylaxis
Source: PLoS One. 2023 Apr 18;18(4):e0284476. doi: 10.1371/journal.pone.0284476 (PMC10112795; doi:10.1371/journal.pone.0284476)

.
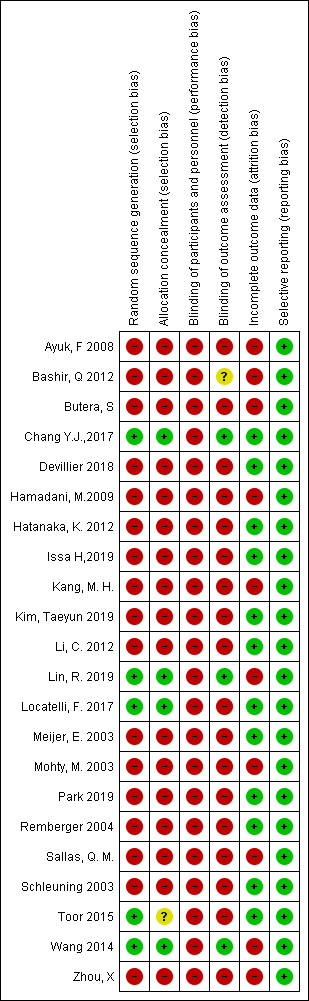


**S1 Fig Risk of bias**

Supplement: S1 Fig — (DOCX) [file pone.0284476.s002.docx]
