# Supplementary material for: Systematic review and meta-analysis of anti-thymocyte globulin dosage as a component of graft-versus-host disease prophylaxis
Source: PLoS One. 2023 Apr 18;18(4):e0284476. doi: 10.1371/journal.pone.0284476 (PMC10112795; doi:10.1371/journal.pone.0284476)

**S2 Fig** Funnel plot for ATG-T

a) II-IV acute GVHD


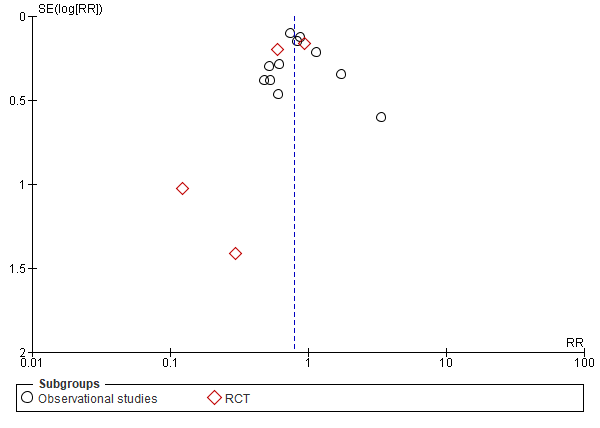


b) III-IV acute GVHD


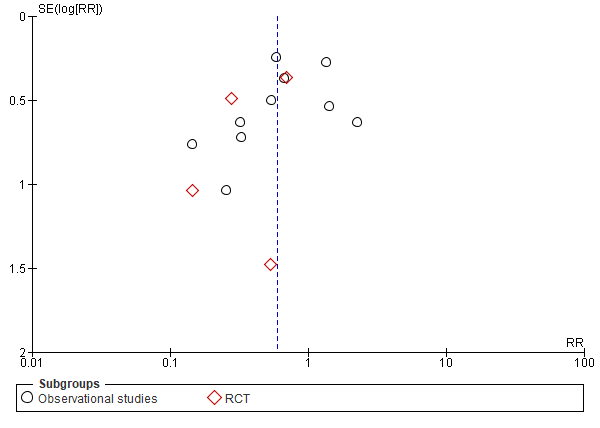


c) Global chronic GVHD


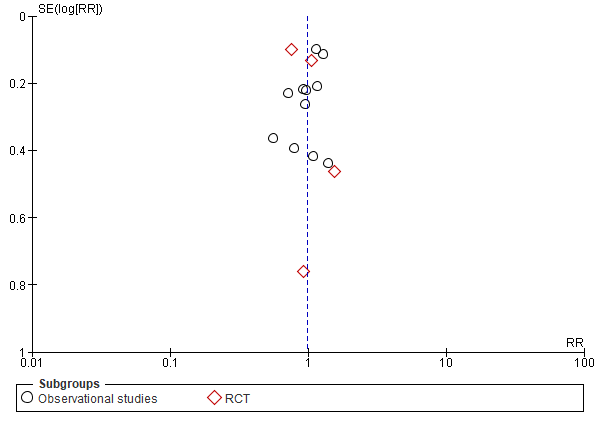


d) Extensive chronic GVHD


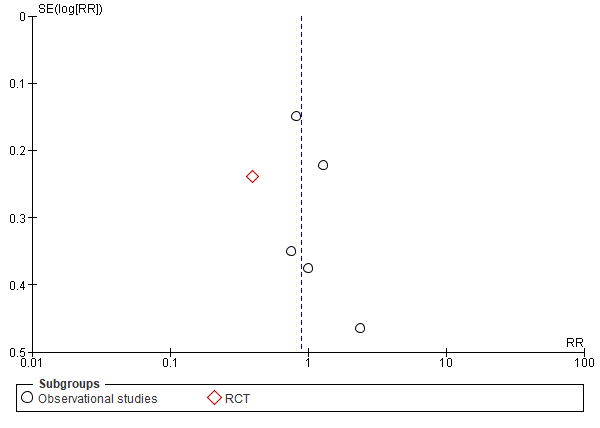


e) Limited chronic GVHD


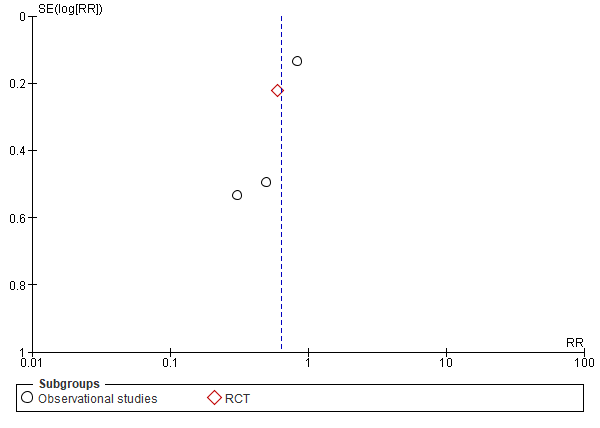


f) CMV reactivation


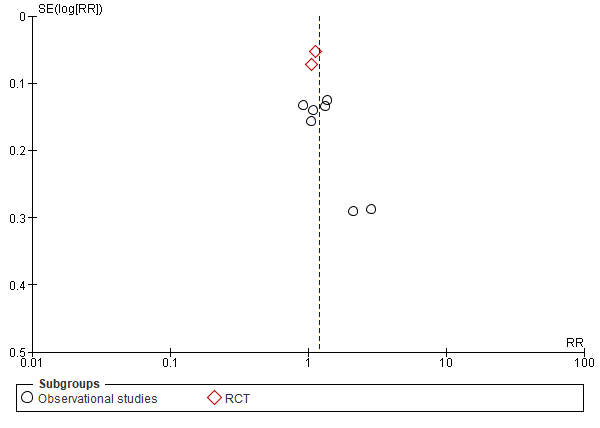


g) EBV reactivation or Lymphoproliferative Disorder Associated to EBV


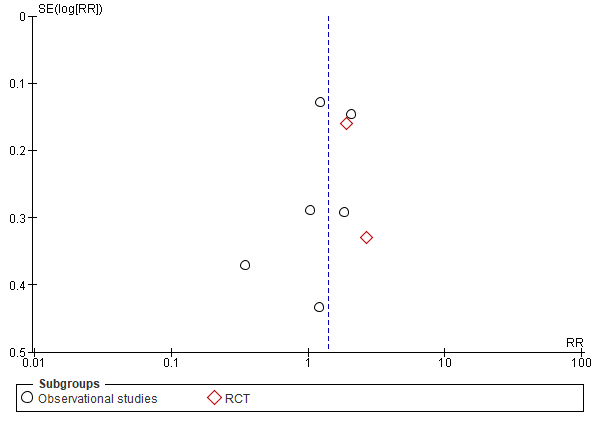

Supplement: S2 Fig — (DOCX) [file pone.0284476.s003.docx]
