# Supplementary material for: Systematic review and meta-analysis of anti-thymocyte globulin dosage as a component of graft-versus-host disease prophylaxis
Source: PLoS One. 2023 Apr 18;18(4):e0284476. doi: 10.1371/journal.pone.0284476 (PMC10112795; doi:10.1371/journal.pone.0284476)
Supplement: S3 Fig — (DOCX) [file pone.0284476.s004.docx]

**S3 Fig** Sensitivity analysis per type of transplantation for ATG-T.

a) II-IV ACUTE GVHD


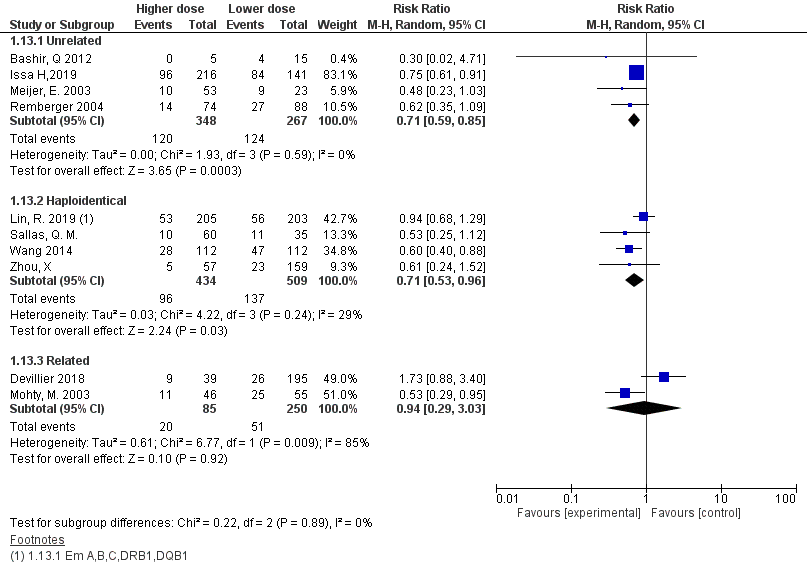


b) III-IV ACUTE GVHD


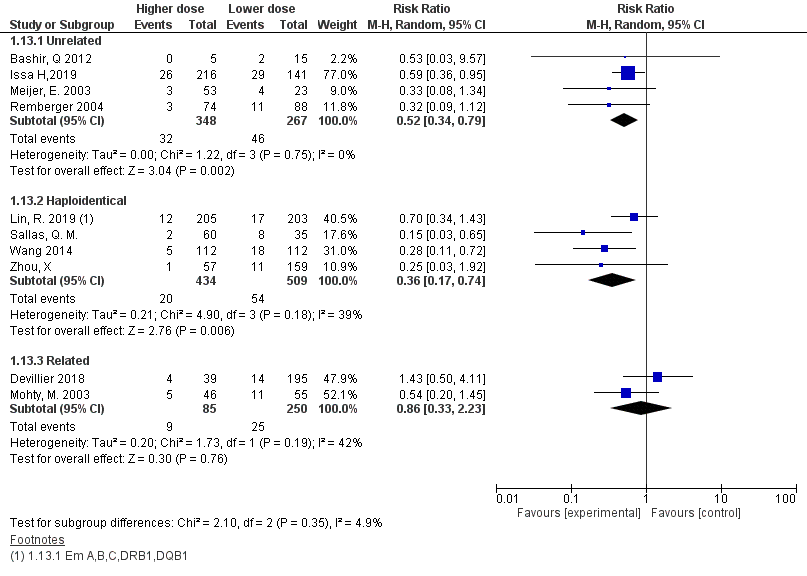


c) GLOBAL CHRONIC GVHD


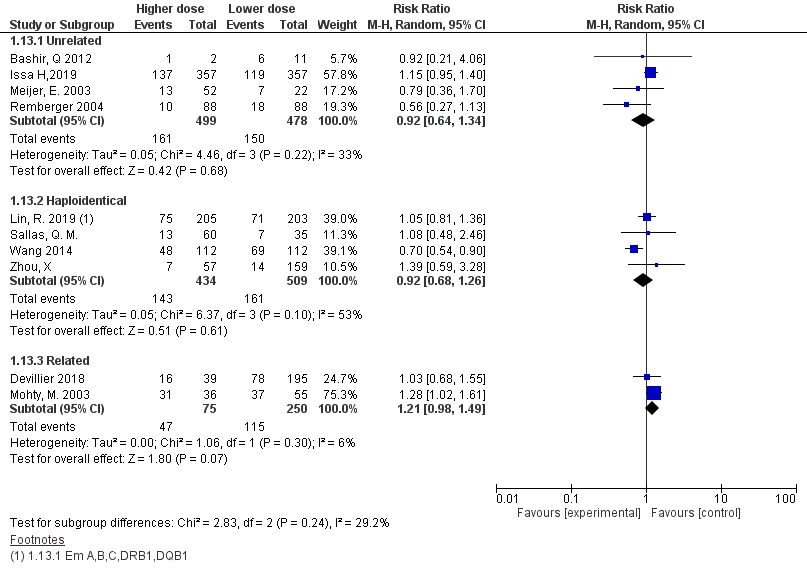


d) Limited chronic GVHD: not enough studies

e) Extensive chronic GVHD: not enough studies

f) CMV reactivation
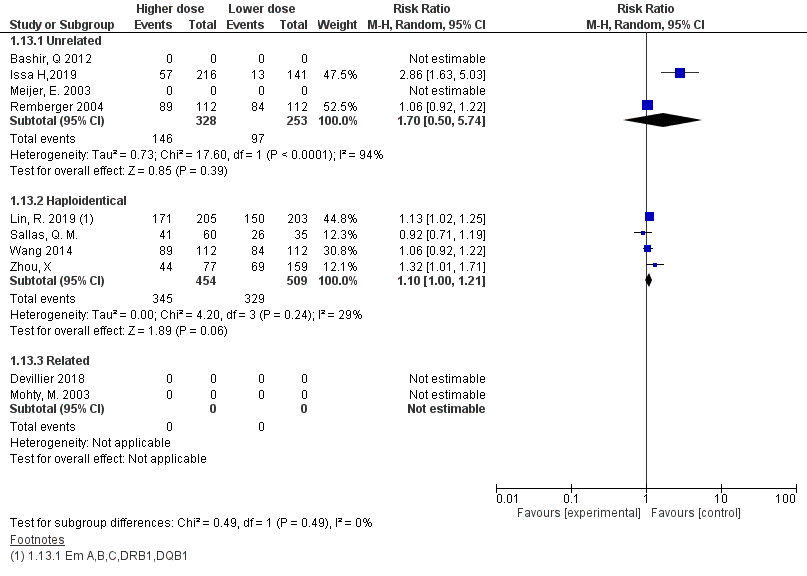


**Fig. 6** Sensitivity analysis per type of transplantation for the comparison between the higher and lower doses of ATG-T in each outcome available. The measure of effect (relative risk) of each study is indicated by blue boxes (size proportional to the weight of the study in the meta-analysis). The lines indicate a 95% confidence interval (95% CIs). The summary of the measure of effect and the 95% confidence interval are indicated by the black diamond.
