# Supplementary material for: Systematic review and meta-analysis of anti-thymocyte globulin dosage as a component of graft-versus-host disease prophylaxis
Source: PLoS One. 2023 Apr 18;18(4):e0284476. doi: 10.1371/journal.pone.0284476 (PMC10112795; doi:10.1371/journal.pone.0284476)
Supplement: S4 Fig — (DOCX) [file pone.0284476.s005.docx]

a) CMV reactivation


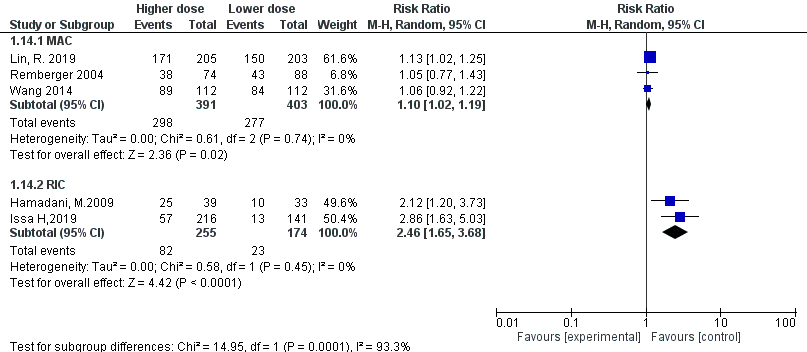


b) EBV reactivation or EBV-associated lymphoproliferative disorder


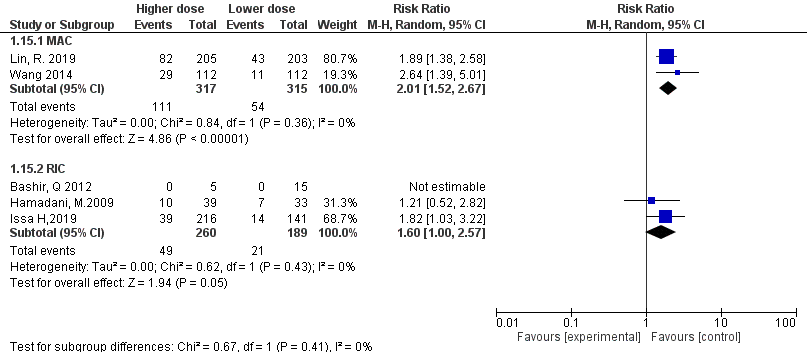


c) II- IV acute GVHD


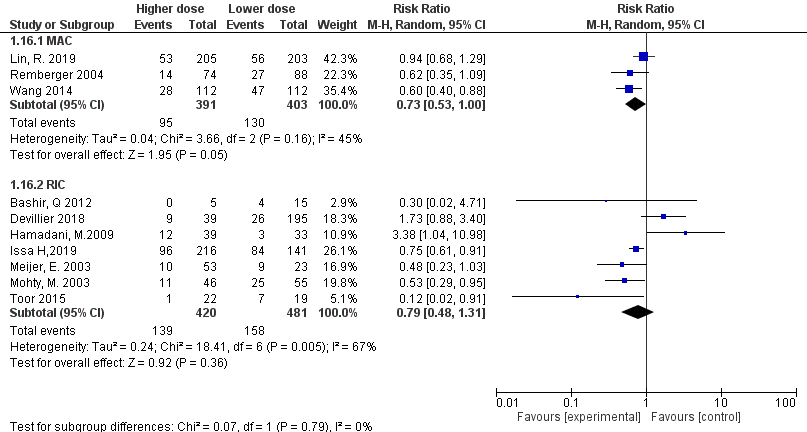


d) III-IV acute GVHD ATG-T


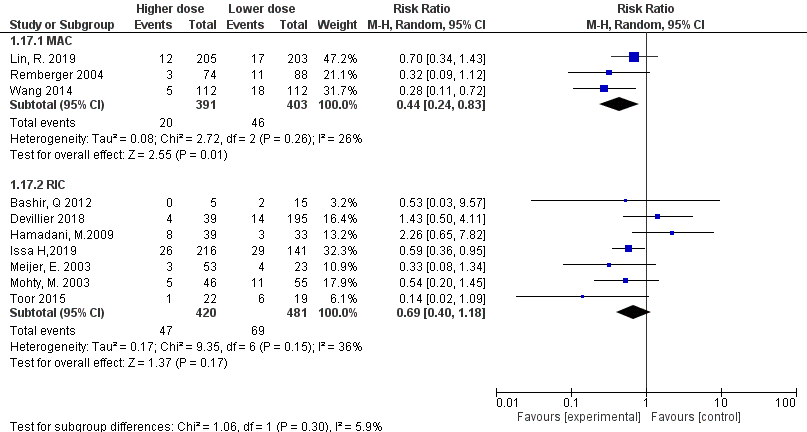


e) Global chronic GVHD ATG-T


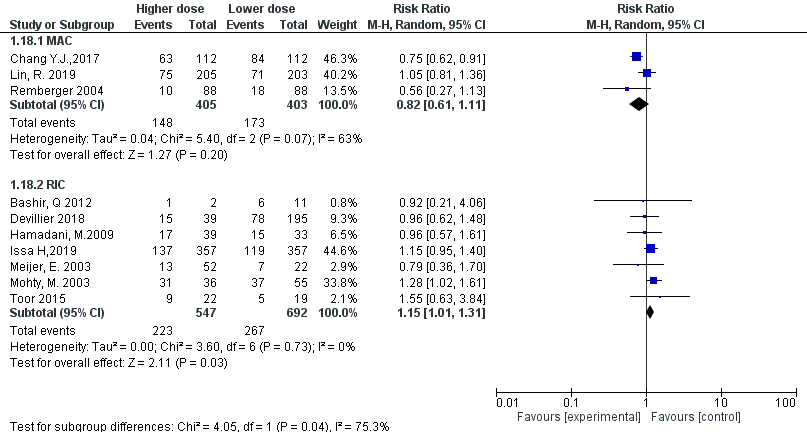


f) Extensive chronic GVHD


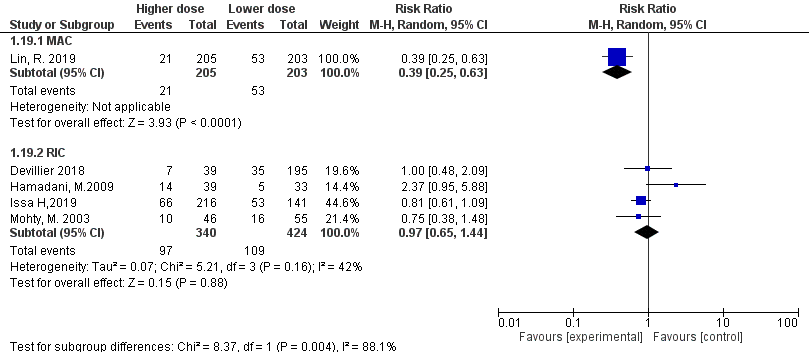


g) Limited chronic GVHD


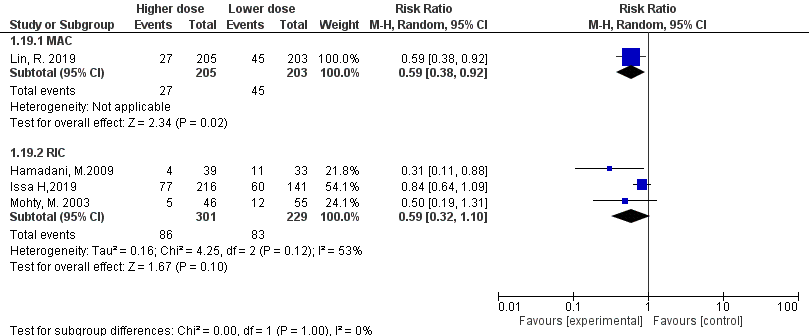


h) Primary or secondary graft failure


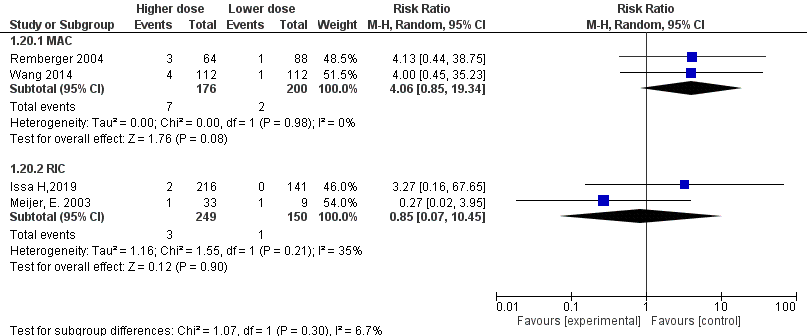


i) Recurrence rate of primary disease (relapse) corrected for 1 year.


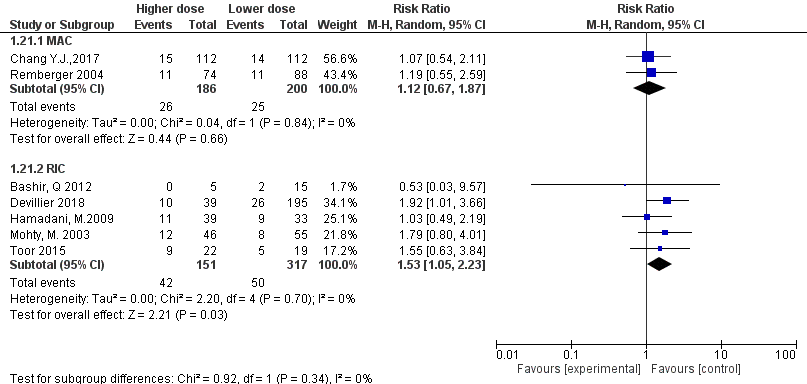


j) Transplant-related mortality corrected for 1 year


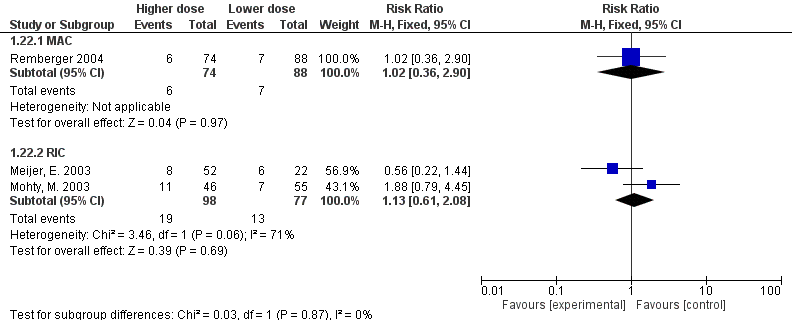


**S4 Fig** Sensitivity analysis per conditioning modality for the comparison between the higher and lower doses of ATG-T in each outcome available. The measure of effect (relative risk) of each study is indicated by blue boxes (size proportional to the weight of the study in the meta-analysis). The lines indicate a 95% confidence interval (95% CIs). The summary of the measure of effect and the 95% confidence interval are indicated by the black diamond.
