# Supplementary material for: Systematic review and meta-analysis of anti-thymocyte globulin dosage as a component of graft-versus-host disease prophylaxis
Source: PLoS One. 2023 Apr 18;18(4):e0284476. doi: 10.1371/journal.pone.0284476 (PMC10112795; doi:10.1371/journal.pone.0284476)
Supplement: S5 Fig — (DOCX) [file pone.0284476.s006.docx]

**S5 Fig** Sensitivity analysis for CMV and EBV, and recurrence.

a) CMV reactivation by type of transplantation


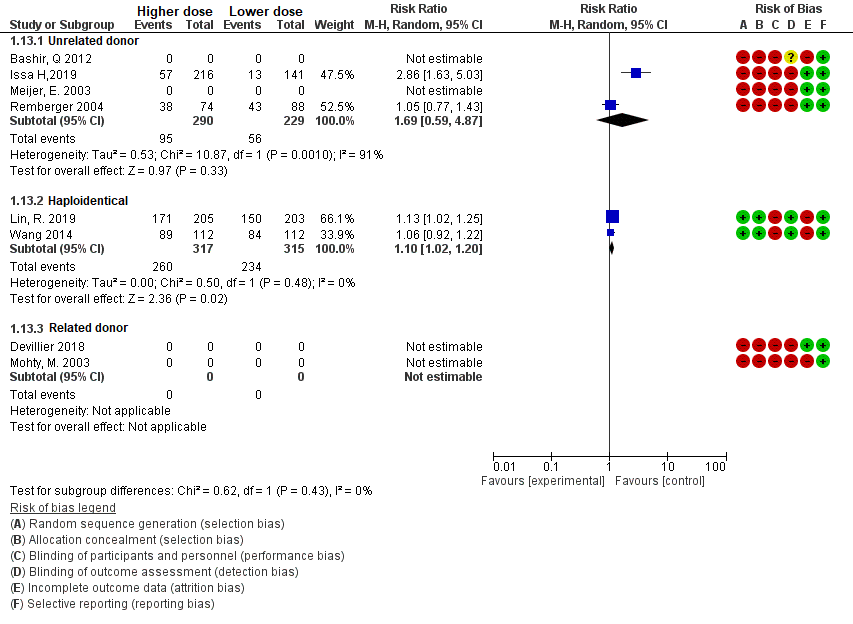


b) CMV reactivation by conditioning modality


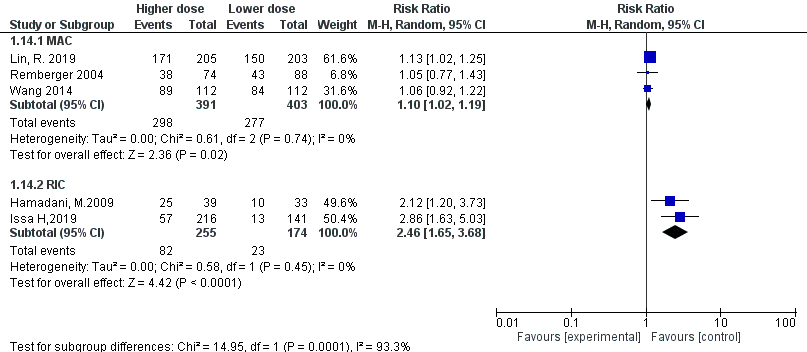


c) EBV reactivation or EBV-associated lymphoproliferative disorder by conditioning modality


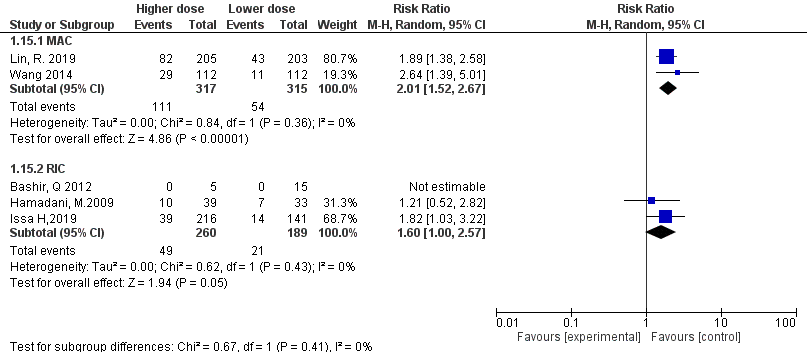


d) Recurrence of primary disease (relapse) corrected for 1 year by conditioning modality


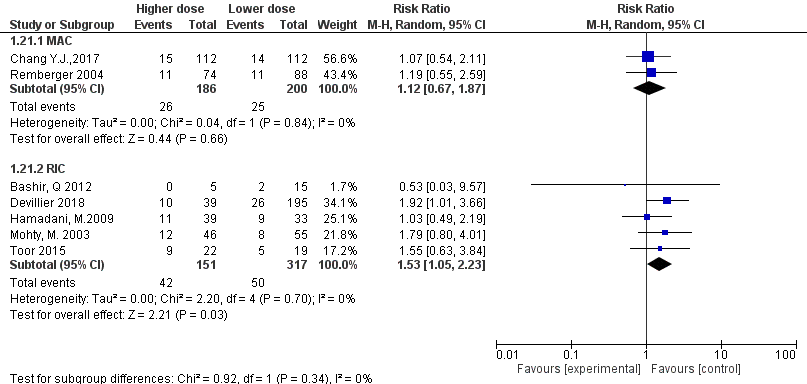


The measure of effect (relative risk) of each study is indicated by blue boxes (size proportional to the weight of the study in the meta-analysis). The lines indicate a 95% confidence interval (95% CIs). The summary of the measure of effect and the 95% confidence interval are indicated by the black diamond
