# Supplementary material for: Systematic review and meta-analysis of anti-thymocyte globulin dosage as a component of graft-versus-host disease prophylaxis
Source: PLoS One. 2023 Apr 18;18(4):e0284476. doi: 10.1371/journal.pone.0284476 (PMC10112795; doi:10.1371/journal.pone.0284476)
Supplement: S6 Fig — (DOCX) [file pone.0284476.s007.docx]

1. II-IV acute GVHD


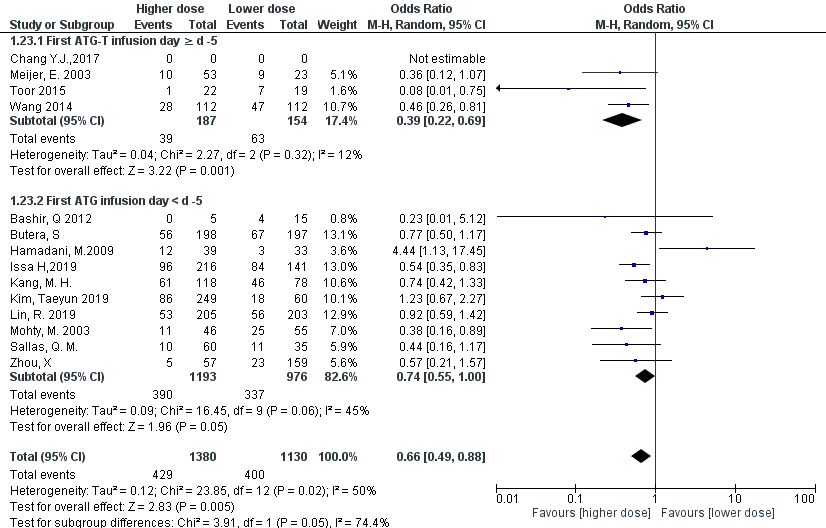


1. III-IV acute GVHD ATG-T


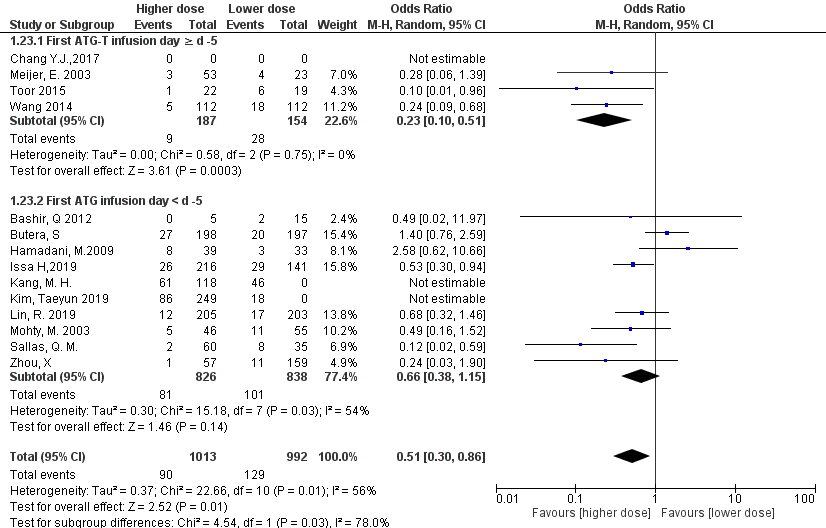


1. Global chronic GVHD ATG-T


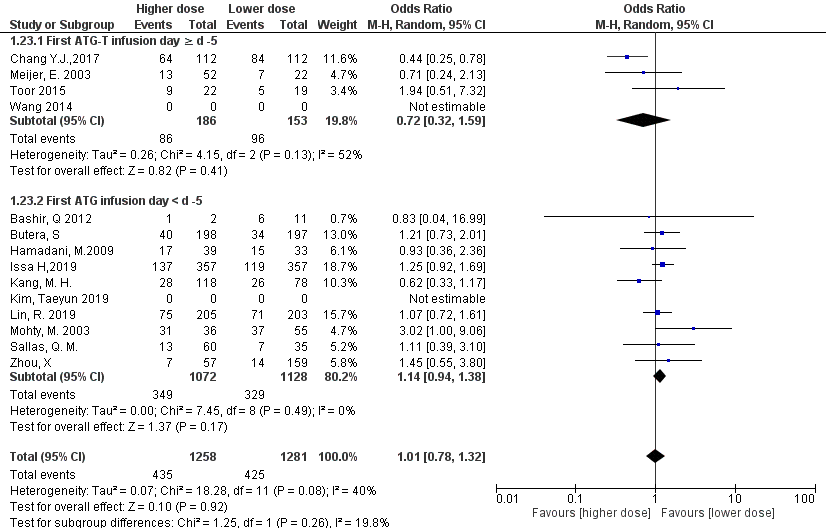


1. CMV reactivation


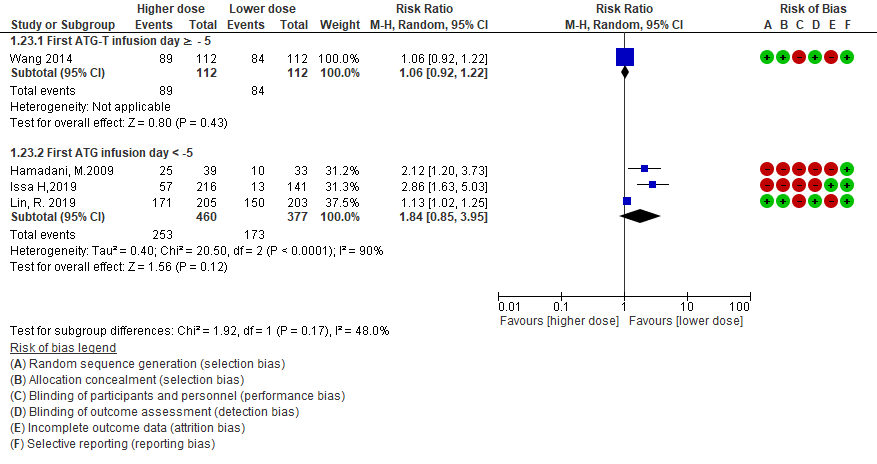


1. EBV reactivation or EBV-associated lymphoproliferative disorder


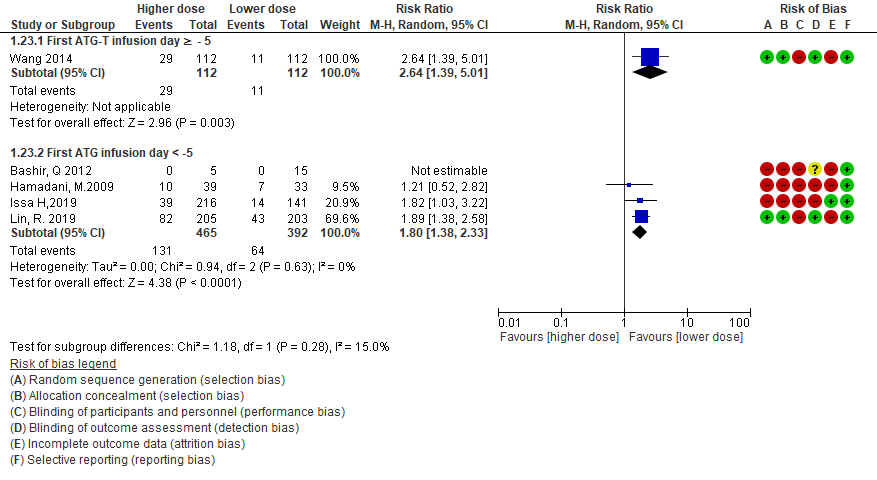


1. Primary or secondary graft failure


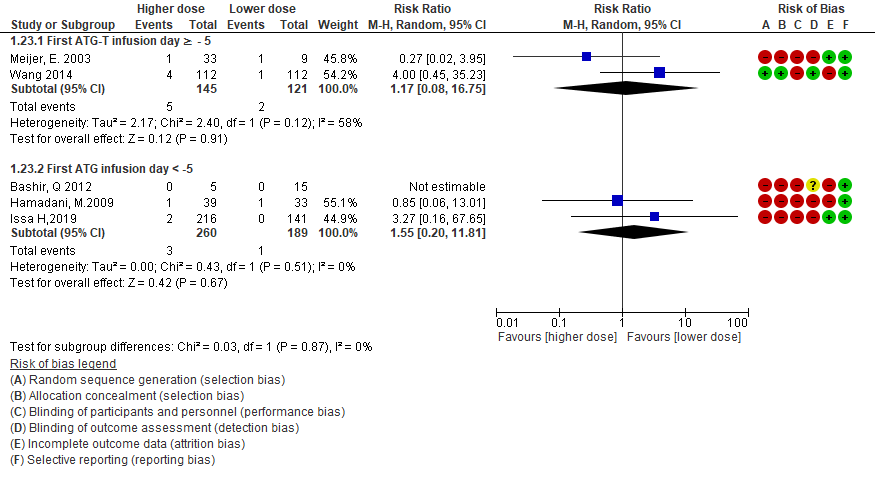


1. Recurrence rate of primary disease (relapse) corrected for 1 year


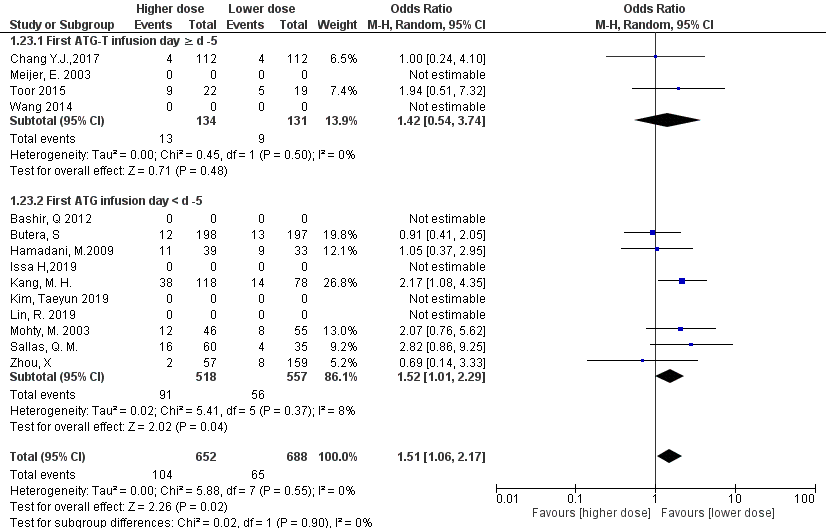


**S6 Fig** Sensitivity analysis per first ATG-T infusion day, before day -5 (≥ -5) or after day -5 (< -5), in each outcome available. The measure of effect (relative risk) of each study is indicated by blue boxes (size proportional to the weight of the study in the meta-analysis). The lines indicate a 95% confidence interval (95% CIs). The summary of the measure of effect and the 95% confidence interval are indicated by the black diamond
