# Supplementary material for: Systematic review and meta-analysis of anti-thymocyte globulin dosage as a component of graft-versus-host disease prophylaxis
Source: PLoS One. 2023 Apr 18;18(4):e0284476. doi: 10.1371/journal.pone.0284476 (PMC10112795; doi:10.1371/journal.pone.0284476)
Supplement: S1 Table — (DOCX) [file pone.0284476.s008.docx]

**S1 Table Search methods and number of studies recovered per database**

| Base | Method | No.studies recovered | Date |
| --- | --- | --- | --- |
| **MEDLINE** | (((("bone marrow transplantation"[mh/Title/Abstract] OR "hematopoietic stem cell transplantation"[mh/Title/Abstract]) OR "haematopoietic stem cell transplantation"[mh/Title/Abstract]) OR "hematopoetic stem cell transplantation"[Title/Abstract]) AND ((("anti thymocyte globulin"[mh/Title/Abstract] OR "antithymocyte globulin"[mh/Title/Abstract] OR Antilymphocyte Globulin[mh/Title/Abstract] OR) OR "antithymocyte immunoglobulin"[mh/Title/Abstract]) OR "antithymocyte antibody"[mh/Title/Abstract])) AND (((("graft versus host disease"[mh/Title/Abstract/mh] OR "graft vs host disease"[Title/Abstract/mh]) OR "graft vs host reaction"[Title/Abstract]) OR "gvh disease"[Title/Abstract]) OR "gvhd"[Title/Abstract])  Filtros: comparative studies; clinical trials | 364  88 | 24/04/2020  01/02/2022 |
| **EMBASE** | (((("bone marrow transplantation":ab,ti OR "hematopoietic stem cell transplantation":ab,ti) OR "haematopoietic stem cell transplantation":ab,ti) OR "hematopoetic stem cell transplantation":ab,ti) AND ((("anti thymocyte globulin":ab,ti OR "antithymocyte globulin":ab,ti) OR "antithymocyte immunoglobulin":ab,ti) OR "antithymocyte antibody":ab,ti)) AND (((("graft versus host disease":ab,ti OR "graft vs host disease":ab,ti) OR "graft vs host reaction":ab,ti) OR "gvh disease":ab,ti) OR "gvhd":ab,ti).  Filtros-> sources: embase; drugs: thymocyte antibody; study types: controlled studies | 113  31 | 23/03/2020  01/02/2022 |
| **LILACS** | Advanced search expression including the terms: bone marrow transplantation OR allogeneic hematopoietic stem cell transplantation AND antithymocyte globulin AND graft-versus-host-disease. In all, the field tw: Text Word (Title + abstract + MeSH / DeCS) is applied | 1  0 | 23/04/2020  01/02/2022 |
| **Cochrane Library** | (" bone marrow transplantation " OR " hematopoietic stem cell transplantation " OR " haematopoietic stem cell transplantation " OR " hematopoetic stem cell transplantation "):ti,ab AND (" anti thymocyte globulin " OR " antithymocyte globulin " OR " antithymocyte immunoglobulin " OR " antithymocyte antibody "):ti,ab AND (" graft versus host disease " OR " graft vs host disease " OR " graft vs host reaction " OR " gvh disease " OR " gvhd "):ti,ab | 55  0 | 23/04/2020  01/02/2022 |
| **Web of Science** | TI = (" bone marrow transplantation " OR " hematopoietic stem cell transplantation " OR " haematopoietic stem cell transplantation " OR " hematopoetic stem cell transplantation ") AND  TI= (" anti thymocyte globulin " OR " antithymocyte globulin " OR " antithymocyte immunoglobulin " OR " antithymocyte antibody ") AND TI= ( " graft versus host disease " OR " graft vs host disease " OR " graft vs host reaction " OR " gvh disease " OR " gvhd ") | 27  27 | 23/04/2020  01/02/2022 |
| **Scielo** | TI = (" bone marrow transplantation " OR " hematopoietic stem cell transplantation " OR " haematopoietic stem cell transplantation " OR " hematopoetic stem cell transplantation ") AND  TI= (" anti thymocyte globulin " OR " antithymocyte globulin " OR " antithymocyte immunoglobulin " OR " antithymocyte antibody ") AND TI= ( " graft versus host disease " OR " graft vs host disease " OR " graft vs host reaction " OR " gvh disease " OR " gvhd ") | 1  0 | 23/04/2020  01/02/2022 |
| **Total** |  | 707 |  |
| **Total after removal of duplicates** |  | 540 |  |
