# Supplementary material for: Systematic review and meta-analysis of anti-thymocyte globulin dosage as a component of graft-versus-host disease prophylaxis
Source: PLoS One. 2023 Apr 18;18(4):e0284476. doi: 10.1371/journal.pone.0284476 (PMC10112795; doi:10.1371/journal.pone.0284476)
Supplement: S1 File — (DOCX) [file pone.0284476.s009.docx]

**Identification of studies via other methods**

**Identification of studies via databases and registers**

Records identified from:

Websites (n =0 )

Organisations (n =0 )

Citation searching (n =6 )

etc.

Records removed *before screening*:

Duplicate records removed (n =167 )

Records marked as ineligible by automation tools (n =0 )

Records removed for other reasons (n = 0)

Records identified from*:

Databases (n =707 )

Registers (n =0 )

**Identification**

Records screened

(n =540 )

Records excluded**

(n =503 )

Reports not retrieved

(n = 0 )

Reports sought for retrieval

(n = 6 )

Reports sought for retrieval

(n =37 )

Reports not retrieved

(n = )

**Screening**

Reports assessed for eligibility

(n = 6)

Reports excluded:

(n = 2 )

.

Reports assessed for eligibility

(n =37 )

Reports excluded:

Wrong comparator (n =12 )

Wrong population (n = 3)

No full text access (n =2 )

Conference abstract (2)

Studies included in review

(n = 22)

Reports of included studies

(n =22 )

**Included**

*Consider, if feasible to do so, reporting the number of records identified from each database or register searched (rather than the total number across all databases/registers).

**If automation tools were used, indicate how many records were excluded by a human and how many were excluded by automation tools.

*From:*  Page MJ, McKenzie JE, Bossuyt PM, Boutron I, Hoffmann TC, Mulrow CD, et al. The PRISMA 2020 statement: an updated guideline for reporting systematic reviews. BMJ 2021;372:n71. doi: 10.1136/bmj.n71. For more information, visit: <http://www.prisma-statement.org/>
